# Automated Workflow for High-Throughput LC–MS/MS Therapeutic Monitoring of Cannabidiol and 7-Hydroxy-cannabidiol in Patients with Epilepsy

**DOI:** 10.3390/ijms26146999

**Published:** 2025-07-21

**Authors:** Michela Palmisani, Francesca Dattrino, Paola Rota, Federica Tacchella, Guido Fedele, Ludovica Pasca, Carlo Alberto Quaranta, Valentina De Giorgis, Thomas Matulli Cavedagna, Chiara Cancellerini, Anna Butti, Gloria Castellazzi, Emilio Russo, Cristina Tassorelli, Pierluigi Nicotera, Valentina Franco

**Affiliations:** 1 Department of Internal Medicine and Therapeutics, University of Pavia, 27100 Pavia, Italy; francesca.dattrino01@universitadipavia.it (F.D.); valentina.franco@unipv.it (V.F.); 2 IRCCS Mondino Foundation, 27100 Pavia, Italy; federica.tacchella@mondino.it (F.T.); ludovica.pasca@mondino.it (L.P.); carloalberto.quaranta01@universitadipavia.it (C.A.Q.); valentina.degiorgis@mondino.it (V.D.G.); cristina.tassorelli@mondino.it (C.T.); pierluigi.nicotera@mondino.it (P.N.); 3 Department of Biomedical, Surgical and Dental Sciences, University of Milan, 20133 Milan, Italy; 4 Institute for Molecular and Translational Cardiology (IMTC), San Donato Milanese, 20097 Milan, Italy; 5 Associazione Farmaceutici dell’Industria (AFI), 20149 Milan, Italy; guido195263@gmail.com; 6 Department of Brain and Behavioral Sciences, University of Pavia, 27100 Pavia, Italy; 7 B.S.N. Srl R&D Laboratory, 26012 Castelleone, Italy; thomas.matulli@bsn-srl.it; 8 Department of Biomedical and Neuromotor Sciences, University of Bologna, 40126 Bologna, Italy; chiara.cancellerini3@unibo.it; 9 Cytiva, 20090 Milan, Italy; anna.butti@cytiva.com; 10 Headache Science and Neurorehabilitation Center, IRCCS Mondino Foundation, 27100 Pavia, Italy; gloria.castellazzi@mondino.it; 11 Department of Electrical, Computer and Biomedical Engineering, University of Pavia, 27100 Pavia, Italy; 12 Science of Health Department, Magna Graecia University, 88100 Catanzaro, Italy; erusso@unicz.it

**Keywords:** automation, LC-MS/MS, CBD, 7-hydroxy-CBD, TDM, epilepsy

## Abstract

This study describes the development and validation of a fully automated workflow serum sample preparation for the quantitative determination of cannabidiol (CBD) and its active metabolite 7-hydroxy-CBD, using liquid chromatography coupled with tandem mass spectrometry (LC-MS/MS). Implemented on an automated platform, the workflow performs key steps such as solvent dispensing, mixing, centrifugation, filtration, and supernatant transfer, producing 96-well plates ready for analysis. Human serum samples were obtained from patients with epilepsy treated with CBD. All samples were processed using both manual and automated methods to evaluate method agreement. Quantification was performed by LC–MS/MS with CBD-*d*_3_ as the internal standard (IS). Method validation was conducted in accordance with European Medicines Agency (EMA) guidelines, confirming that the automated protocol meets the recommended acceptance criteria for both intraday and interday precision and accuracy. Calibration curves demonstrated excellent linearity across the concentration ranges. Comparative analysis using Passing–Bablok regression and Bland–Altman plots demonstrated strong agreement between the methods. These findings support the clinical applicability of the automated method for the therapeutic drug monitoring (TDM) of CBD and 7-hydroxy-CBD, and its robust performance and scalability provide a solid foundation for the development of an expanded analytical panel covering a broader range of antiseizure medications (ASMs), enabling more standardized TDM protocols in clinical practice.

## 1. Introduction

Cannabidiol (CBD) is a non-euphoric phytocannabinoid that exerts several beneficial pharmacological effects. The compound has analgesic and anti-inflammatory activities mediated by inhibiting cyclooxygenase and lipoxygenase. This anti-inflammatory action is several hundred times more potent than acetylsalicylic acid [1]. Several investigations have confirmed CBD’s anxiolytic, antiemetic, antipsychotic, neuroprotective, and antiseizure properties [2].

Ongoing research is exploring its potential use in the treatment of psychiatric disorders, neurodegenerative conditions, and inflammation-related diseases, while it has been approved by the U.S. Food and Drug Administration and the European Medicines Agency (EMA) in 2018 and 2019, respectively, as an adjunctive therapy for refractory seizures associated with Dravet syndrome, Lennox–Gastaut syndrome, and tuberous sclerosis complex in a purified pharmaceutical-grade oral formulation [3,4].

Therapeutic drug monitoring (TDM) represents a crucial clinical tool for the individualized management of antiseizure medications (ASMs), aiming to optimize therapeutic efficacy while minimizing adverse effects [5]. TDM of ASMs is based on precise quantification of drug concentrations in plasma or serum, enabling dose optimization by accounting for inter-individual pharmacokinetic variability, drug–drug interactions, and patient adherence patterns [6]. Liquid chromatography coupled with tandem mass spectrometry (LC–MS/MS) has become the analytical standard in TDM owing to its high analytical sensitivity, specificity, and robustness. Although recent advancements in chromatographic methodologies have significantly increased analytical throughput, sample preparation continues to represent a major limiting step, especially within high-throughput analytical workflows. The predominant sample preparation approaches for plasma-based LC–MS/MS assays include protein precipitation (PP), solid-phase extraction (SPE), and liquid–liquid extraction (LLE) [7,8,9,10,11,12,13,14,15,16,17,18,19,20]. Among these, PP is frequently employed due to its procedural simplicity, compatibility with automation, and suitability for 96-well plate workflows. However, manual execution of PP is labor-intensive, prone to inter-operator variability, and limits reproducibility in large-scale analyses. To overcome these limitations, the automation of pre-analytical workflows has emerged as a key innovation in bioanalytical laboratories. Integrated robotic platforms capable of performing liquid handling, centrifugation, filtration, and plate management have been shown to enhance throughput, improve data reproducibility, and reduce operator-dependent variability. Although several studies have reported the quantification of CBD and its metabolites in different biological matrices using LC–MS/MS [16,21,22,23,24,25,26,27,28,29,30,31,32,33,34,35], to date no fully automated sample preparation protocol has been described for the simultaneous quantification of CBD and its pharmacologically active metabolite, 7-hydroxy-CBD, in human serum.

The aim of this study was to develop and validate a fully automated sample preparation method for the quantitative determination of CBD and its major active metabolite 7-hydroxy-CBD in human serum, employing LC–MS/MS for downstream analysis. Method performance was assessed in comparison to a previously established manual protocol [36] using Passing–Bablok regression and Bland–Altman analysis to evaluate concordance. The analytical protocol was further applied to clinical serum samples from patients receiving CBD as part of their ASM regimen. The proposed method supports the broader implementation of automation in clinical bioanalysis, with direct implications for improving the efficiency and reliability of TDM of ASMs.

## 2. Results

Intraday and interday precision and accuracy for both methods are reported in Table 1, Table 2, Table 3 and Table 4. All the results obtained complied with EMA guidelines [37]. The concentrations of analytes are reported as ng/mL and expressed as ± standard deviation (SD).

### 2.1. Manual Method

For CBD and 7-hydroxy-CBD in serum intraday and interday precision, measured across four curves ranged between 1% and 6.6% and 1.3% and 7.9%, respectively. Accuracy in serum ranged from 92.5% to 111.8% for CBD and from 92.7% to 105.1% for 7-hydroxy-CBD (Table 1 and Table 2). Extraction recoveries for CBD and 7-hydroxy-CBD (n = 5) in serum ranged from 80% to 85% and 86% to 92%, respectively.

**Table 1:** Intraday and interday precision and accuracy of CBD with the manual sample preparation.

Spiked Concentrations (ng/mL)	Measured (ng/mL)	Precision (%)	Accuracy (%)	Spiked Concentrations (ng/mL)	Measured (ng/mL)	Precision (%)	Accuracy (%)
Serum CBD	Intraday			Serum CBD	Interday		
2.5 (LOQ) (n = 5)	2.8 ± 0.1	4.5	111.8	2.5 (LOQ) (n = 10)	2.8 ± 0.2	6.3	110.5
35 (QC1) (n = 5)	35.3 ± 1	2.7	100.8	35 (QC1) (n = 20)	34.2 ± 2.3	6.6	97.8
350 (QC2) (n = 5)	327.6 ± 18.4	5.6	93.6	350 (QC2) (n = 20)	336.4 ± 21	6.2	96.1
750 (QC3) (n = 5)	693.6 ± 7	1	92.5	750 (QC3) (n = 20)	735.2 ± 41.2	5.6	98

*LOQ, limit of quantification; QC1, low-concentration quality control; QC2, medium-concentration quality control; QC3, high-concentration quality control.*

**Table 2:** Intraday and interday precision and accuracy of 7-hydroxy-CBD with the manual sample preparation.

Spiked Concentrations (ng/mL)	Measured (ng/mL)	Precision (%)	Accuracy (%)	Spiked Concentrations (ng/mL)	Measured (ng/mL)	Precision (%)	Accuracy (%)
Serum 7-Hydroxy-CBD	Intraday			Serum 7-Hydroxy-CBD	Interday		
5 (LOQ) (n = 5)	4.8 ± 0.3	6.5	95.4	5 (LOQ) (n = 10)	4.6 ± 0.4	7.8	92.7
17.5 (QC1) (n = 5)	18.4 ± 0.4	2	105.1	17.5 (QC1) (n = 20)	17.5 ± 1.4	7.9	100.1
175 (QC2) (n = 5)	164.8 ± 4.2	2.6	94.2	175 (QC2) (n = 20)	165.4 ± 11.9	7.2	94.5
375 (QC3) (n = 5)	354 ± 4.7	1.3	94.4	375 (QC3) (n = 20)	360.6 ± 24.5	6.8	96.1

*LOQ, limit of quantification; QC1, low-concentration quality control; QC2, medium-concentration quality control; QC3, high-concentration quality control.*

**Figure 1:**
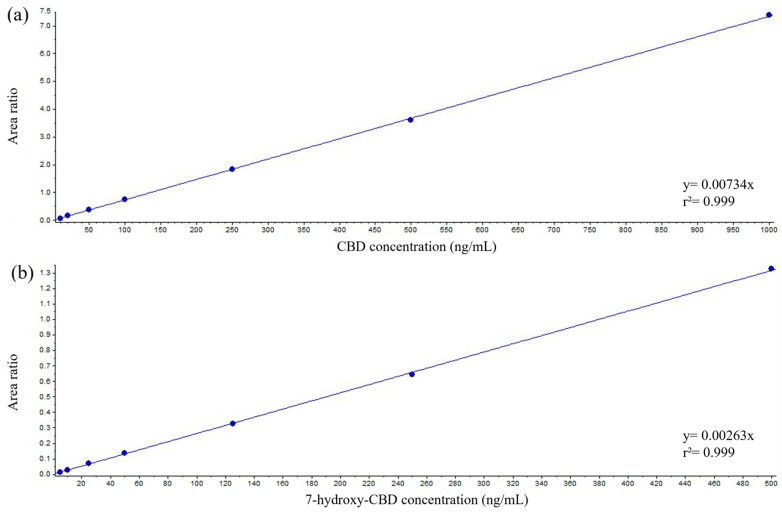
Representative curves of CBD (**a**) and 7-hydroxy-CBD (**b**) in serum (manual sample preparation).

Calibration curves exhibited linearity across the tested concentration range in serum for both analytes (Figure 1a,b).

### 2.2. Automated Method

For CBD and 7-hydroxy-CBD in serum, intraday and interday precision, measured in four curves, ranged from 1.5% to 11.5% and 2.4% and 8.1%, respectively. Accuracy values in serum ranged from 87.9% to 109.3% for CBD and 91.9% to 103% for 7-hydroxy-CBD (Table 3 and Table 4). Extraction recoveries in serum for CBD and 7-hydroxy-CBD (n = 5) ranged between 80% and 104% and 81% and 92%, respectively.

**Table 3:** Intraday and interday precision and accuracy of CBD in serum with the automated sample preparation.

Spiked Concentrations (ng/mL)	Measured (ng/mL)	Precision (%)	Accuracy (%)	Spiked Concentrations (ng/mL)	Measured (ng/mL)	Precision (%)	Accuracy (%)
Serum CBD	Intraday			Serum CBD	Interday		
2.5 (LOQ) (n = 5)	2.6 ± 0.3	11.5	105.3	2.5 (LOQ) (n = 10)	2.7 ± 0.2	8.4	109.3
35 (QC1) (n = 5)	30.8 ± 1	3.4	87.9	35 (QC1) (n = 20)	32.6 ± 2.1	6.3	93.2
350 (QC2) (n = 5)	308.4 ± 4.7	1.5	88.1	350 (QC2) (n = 20)	329.9 ± 20.1	6.1	94.3
750 (QC3) (n = 5)	688 ± 13.6	2	91.7	750 (QC3) (n = 20)	718.3 ± 36.9	5.1	95.8

*LOQ, limit of quantification; QC1, low-concentration quality control; QC2, medium-concentration quality control; QC3, high-concentration quality control.*

**Table 4:** Intraday and interday precision and accuracy of 7-hydroxy-CBD in serum with the automated sample preparation.

Spiked Concentrations (ng/mL)	Measured (ng/mL)	Precision (%)	Accuracy (%)	Spiked Concentrations (ng/mL)	Measured (ng/mL)	Precision (%)	Accuracy (%)
Serum 7-Hydroxy-CBD	Intraday			Serum 7-Hydroxy-CBD	Interday		
5 (LOQ) (n = 5)	4.6 ± 0.3	6.6	91.9	5 (LOQ) (n = 10)	4.8 ± 0.4	7.8	95.8
17.5 (QC1) (n = 5)	16.7 ± 0.7	4.3	95.4	17.5 (QC1) (n = 20)	18 ± 1.5	8.1	103
175 (QC2) (n = 5)	174.4 ± 4.2	2.4	99.7	175 (QC2) (n = 20)	175.6 ± 9.6	5.4	100.4
375 (QC3) (n = 5)	356.6 ± 16.7	4.7	95.1	375 (QC3) (n = 20)	374 ± 24.3	6.5	99.7

*LOQ, limit of quantification; QC1, low-concentration quality control; QC2, medium-concentration quality control; QC3, high-concentration quality control.*

**Figure 2:**
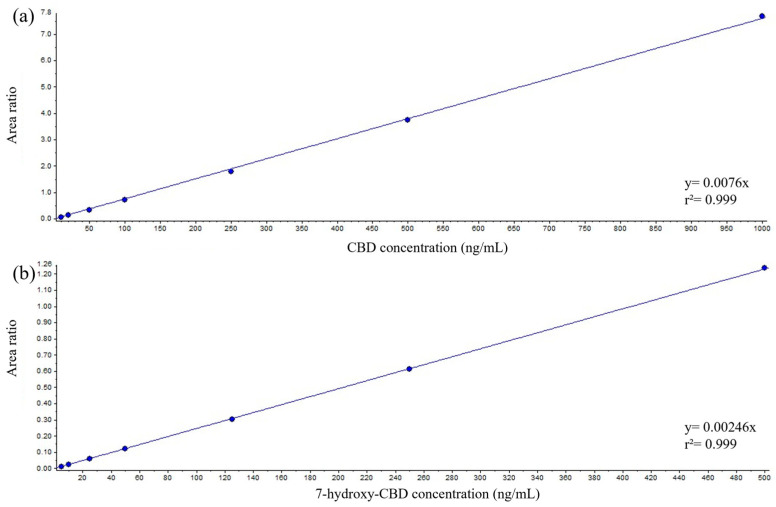
Representative curves of CBD (**a**) and 7-hydroxy-CBD (**b**) in serum (automated sample preparation).

Calibration curves exhibited linearity across the tested concentration range in serum for both analytes (Figure 2a,b). 

### 2.3. Matrix Effect

No significant matrix effect was observed, with intraday and interday precisions below 4.9% for both analytes and accuracy ranging from 95.7% to 104% for CBD and from 98.1% to 105.9% for 7-hydroxy-CBD.

### 2.4. Analysis of Patient Samples and Comparison of Assay Performance Between Manual and Automated Sample Preparation Techniques

**Figure 3:**
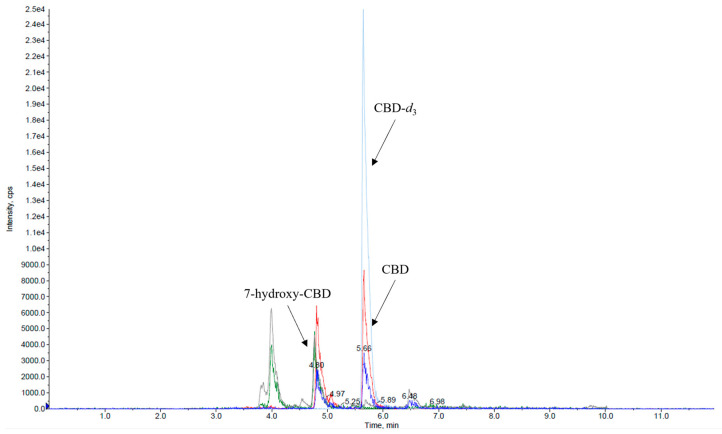
Representative chromatogram obtained from a serum sample processed using the manual preparation method, showing CBD and 7-hydroxy-CBD at concentrations of 49 ng/mL and 81 ng/mL, respectively. CBD (red line); 7-hydroxy-CBD (grey line); CBD-*d*_3_ (light blue line).

**Figure 4:**
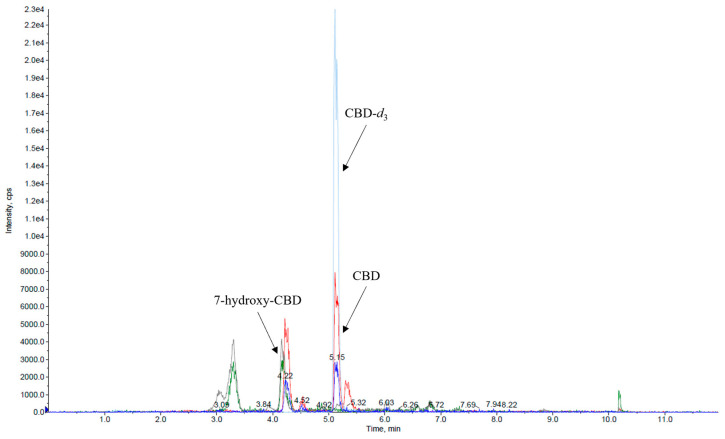
Representative chromatogram obtained from a serum sample processed using the automated preparation method, with measured concentrations of CBD and 7-hydroxy-CBD at 49 ng/mL and 80 ng/mL, respectively. CBD (red line); 7-hydroxy-CBD (grey line); CBD-*d*_3_ (light blue line).

Clinical parameters and results of CBD and 7-hydroxy-CBD quantification in serum from six different subjects with Lennox–Gastaut syndrome are reported in Table 5. CBD concentration in serum ranged from 15 to 254 ng/mL and from 14 to 253 ng/mL, across patients, for manual and automated sample preparation method, respectively. 7-Hydroxy-CBD concentration ranged from 12 to 150 ng/mL and from 13 to 147 ng/mL, across patients, for manual and automated sample preparation method, respectively. Figure 3 and Figure 4 show representative chromatograms of CBD and 7-hydroxy-CBD in serum obtained with manual and automated sample preparation, respectively, from a subject with Lennox–Gastaut syndrome, treated with CBD at a dose of 11.1 mg/kg/day.

**Table 5:** CBD and 7-hydroxy-CBD levels in serum of the subjects with Lennox–Gastaut syndrome included in the analysis. Each concentration value represents the mean of two measurements per sample.

		Manual		Automated	
Patient Number	Dose of CBD (mg/kg/day)	CBD Concentration (ng/mL)	7-Hydroxy-CBD Concentration (ng/mL)	CBD Concentration (ng/mL)	7-Hydroxy-CBD Concentration (ng/mL)
1	5	15	12	14	13
2	8.7	36	78	33	79
3	10.6	39	52	38	53
4	11.1	49	81	49	80
5	17.9	254	150	253	147
6	8.9	22	20	20	19

**Figure 5:**
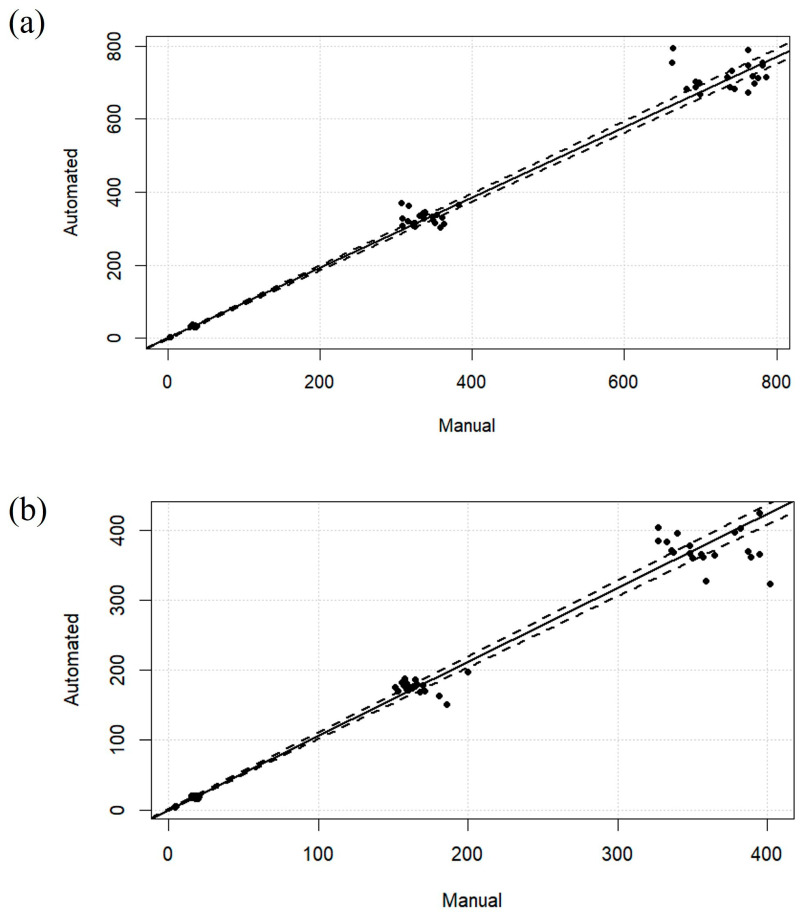
Passing–Bablok regression plots showing the correlations of CBD (**a**) and 7-hydroxy-CBD (**b**) concentrations, reported in ng/mL, between the manual and automated methods (n = 70 pairs), with dotted lines representing the 95% CIs.

**Figure 6:**
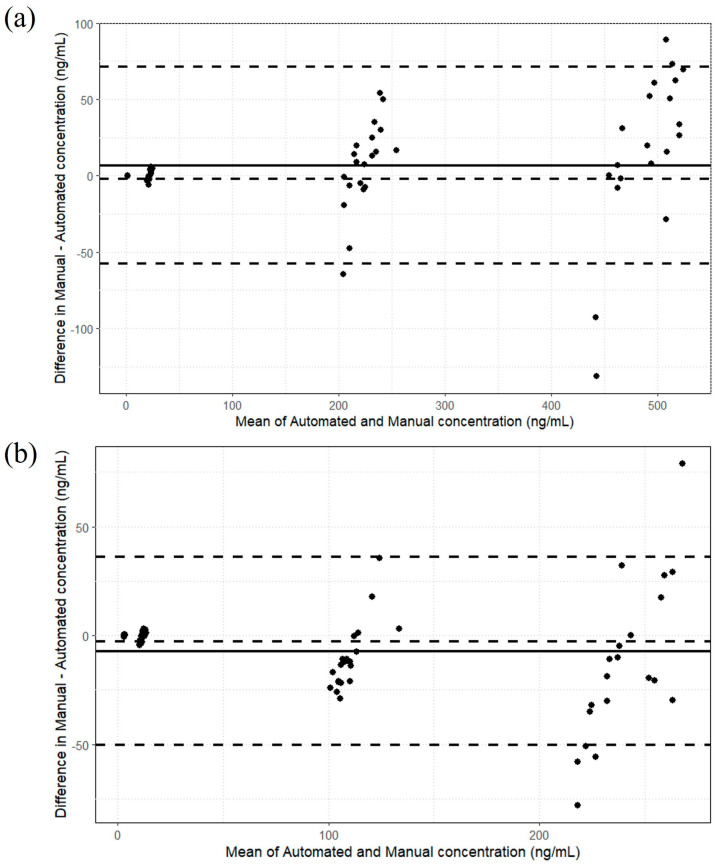
Bland–Altman plots comparing CBD (**a**) and 7-hydroxy-CBD (**b**) concentrations obtained from serum samples prepared using manual versus automated methods (n = 70 pairs). The x-axis represents the mean concentration of each pair, while the y-axis indicates the difference between the two preparation methods. For CBD, the mean bias was 7.1316 ng/mL (95% CI: −57.3373 to 71.6004). The lower limit of agreement (LoA) (95% CI) was −0.7113 ng/mL (−70.9216 to −43.7530), and the upper LoA (95% CI) was 14.9745 ng/mL (58.0162 to 85.1847). For 7-hydroxy-CBD, the mean bias was −6.9539 ng/mL (95% CI: −50.0299 to 36.1221), with a lower LoA (95% CI) of −12.1942 ng/mL (95% CI: −59.1064 to −40.9533) and an upper LoA (95% CI) of −1.7135 ng/mL (95% CI: 27.0456 to 45.1987). The solid line denotes the mean bias, while the dotted lines represent the LoA. A fitted regression line with its 95% CI is also included.

Figure 5a,b present the analytical results for CBD and 7-hydroxy-CBD, respectively, obtained via Passing–Bablok regression analysis applied to limit of quantification (LOQ) and quality control (QC) serum samples. The regression analysis indicated no significant bias or proportional errors. For CBD, the regression curve yielded an intercept of 0.0001 (95% confidence interval (CI): −1.0161 to 0.6635) and a slope of 0.9642 (95% CI: 0.9387 to 0.9897). For 7-hydroxy-CBD, the intercept and slope were −0.1362 (95% CI: −0.7639 to 0.7930), and of 1.0595 (95% CI: 1.0217 to 1.0914), respectively. Lin’s concordance correlation coefficients were 0.9935 for CBD and 0.9879 for 7-hydroxy-CBD, indicating excellent agreement between the two methods. The cumulative sum test returned *p*-values significantly above the threshold for statistical significance (*p* > 0.4 for CBD and *p* > 0.2 for 7-hydroxy-CBD), confirming no substantial difference between the manual and automated sample preparation methods. Furthermore, Bland–Altman analysis revealed minimal discrepancies in the concentration measurements between samples prepared by manual and automated methods, with only five samples for CBD and 7-hydroxy-CBD exceeding the predefined tolerance limits (Figure 6a,b).

## 3. Discussion and Conclusions

CBD has been the focus of extensive bioanalytical investigations in recent years, with several studies reporting its quantification across a wide spectrum of biological matrices, including blood, plasma, oral fluid, urine, hair, aqueous humor and exhaled breath [16,21,22,23,24,25,26,27,28,29,30,31,32,33,34,35]. Despite this broad analytical coverage, to the best of our knowledge, no previous work has implemented a fully automated sample preparation protocol for the LC–MS/MS quantification of CBD and its major active metabolite, 7-hydroxy-CBD, in serum.

In this study, we have developed and validated a high-throughput, automated sample preparation workflow, integrated within a robotic platform, which streamlines all critical pre-analytical phases. Compared to previously reported LC–MS/MS methods for the quantification of CBD and its metabolites, which frequently rely on manual extraction techniques such as LLE or SPE and often require larger sample volumes (100–500 µL), the proposed method offers a significantly streamlined and miniaturized workflow. It requires only 75 µL of serum per sample and adheres to the performance requirements set forth by the EMA for bioanalytical method validation [37]. While conventional approaches remain effective, they are labor-intensive, less scalable, and susceptible to operator-dependent variability. In contrast, the proposed automated workflow processes 96 samples in parallel, substantially reducing turnaround time and improving analytical reproducibility by eliminating manual handling. Importantly, the workflow includes a filtration-based sample purification step by using the Positive Pressure Unit PPU V4 and Acroprep™ Advance 30K Omega filtration plate, which obviates the need for online SPE. The resulting extract is of sufficient clarity and quality to allow for direct LC-MS/MS injection without compromising sensitivity or selectivity, as demonstrated by the minimal matrix effects observed and the excellent reproducibility of the results. Furthermore, the automated method’s performance was supported through direct comparison with a previously published manual method [36], demonstrating high concordance between the two approaches, as confirmed by Passing–Bablok regression and Bland–Altman plots. Although the initial implementation of robotic systems may require specialized instrumentation and setup, the benefits in terms of throughput, standardization, and reduced human error outweigh this limitation in high-demand laboratory environments.

Given its efficiency, accuracy, and reproducibility, the automated procedure developed in this study is suitable for implementation in TDM of CBD and its active 7-hydroxy metabolite. The high-throughput capability, minimal sample volume requirement, and reduced operator involvement make it adequate for both routine clinical laboratory workflows and clinical research settings. Moreover, the automated platform offers significant potential for adaptation to other matrices and ASMs, allowing for the processing of several compounds within a unified protocol. This opens new avenues for application in pharmacokinetic studies, including those involving pediatric and vulnerable patient populations where standardization and volume minimization are critical.

## 4. Materials and Methods

### 4.1. Standards and Chemicals

Reference standards for CBD and its deuterated analog, CBD-*d_3_*, were obtained from Merck (Merck KGaA, Darmstadt, Germany). The metabolite 7-hydroxy-CBD was obtained from Toronto Research Chemicals (TRC, Toronto, ON, Canada). Ultrapure water used for solution and eluent preparation was produced with a Millipore-Q-plus system (Millipore, Milan, Italy). LC-MS grade acetonitrile, methanol, isopropanol, formic acid (99%), and ammonium formate (99%), used for preparing the mobile phase, were supplied by VWR International (VWR International, Radnor, PA, USA). The 96-well Acroprep™ Advance 30K Omega filtration plates (Part number 8035) were purchased from Cytiva (Cytiva, Milan, Italy). Drug-free serum used in the preparation of calibrators and QC samples, was obtained from healthy adult donors who provided written informed consent.

### 4.2. Automated Platform and LC-MS Equipment

**Figure 7:**
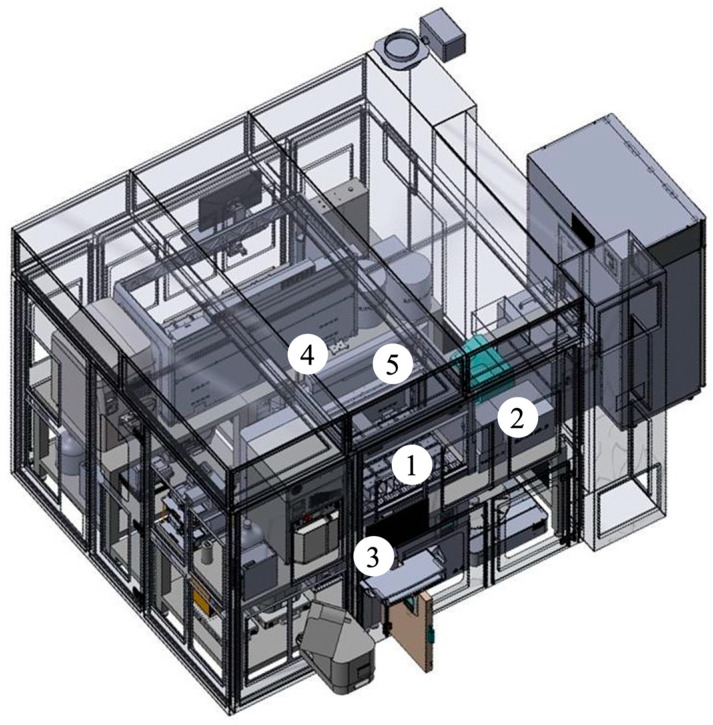
Schematic representation of the high-throughput automated sample preparation platform: (1) Biomek i7 hybrid workstation; (2) positive pressure unit; (3) microplate centrifuge; (4) robotic arm; (5) ambient storage unit.

**Figure 8:**
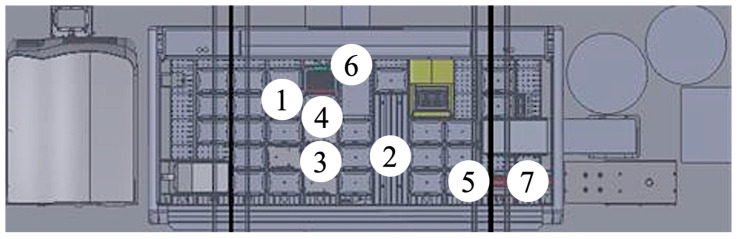
Schematic layout of the Biomek i7 deck configuration: (1) tip racks; (2) source labware; (3) 96-well elution plate; (4) solvent reservoir and centrifuge balance; (5) 96-well filtration plate positioned for integration with the positive pressure unit; (6) Biomek i7 servo shuttle system for plate positioning; and (7) moving tray enabling automated labware transfer to the positive pressure unit module.

Quantification was performed using an LC-MS/MS setup consisting of a 3200 QTRAP^®^ triple quadrupole linear ion trap mass spectrometer with a TurboIonSpray interface (Applied Biosystems Sciex, Darmstadt, Germany), coupled to an ExionLC 100 High-Performance Liquid Chromatography (HPLC) system. The HPLC unit included a quaternary low-pressure mixing pump, column oven, autosampler, degasser, and system controller (Applied Biosystems Sciex, Darmstadt, Germany). Chromatographic separation was conducted on a monolithic C18 column Onyx, 100 × 3 mm i.d. (Phenomenex, Bologna, Italy) maintained at 25 °C. The online SPE (manual workflow) was carried out with perfusion column POROS R1, 2.1 × 30 mm i.d., 20 μm (B.S.N. Srl R&D Laboratory, Castelleone, Italy). The LC–MS/MS parameters applied in this study were adopted from the previously published and fully validated method described by Franco et al. (2022) [36]. The development of the automated sample preparation protocol was implemented on a high-throughput Biomek SAMI EX Workstation (Beckman Coulter Life Sciences, Indianapolis, IN, USA) as the central liquid-handling system. The system integrates the following: (1) a Biomek i7 hybrid workstation (Beckman Coulter Life Sciences, Indianapolis, IN, USA) for liquid handling operations; (2) a Positive Pressure Unit PPU V4 (Amplius GmbH, Rostock, Germany) for SPE under controlled pressure; (3) a microplate centrifuge (Agilent Technologies, Waldbronn, Germany) for efficient pellet separation; (4) a SCARA robotic arm for plate transfer and coordination across modules; and (5) a Cytomat 10 ambient storage unit (Thermo Fisher Scientific, Cleveland, OH, USA) for labware storage (Figure 7). The detailed layout of the Biomek i7 deck is depicted in Figure 8. In particular the workstation is equipped with (1) tip racks for disposable pipette tips; (2) source labware containing calibrators, QCs, internal standard (IS) solutions, and patient serum samples; (3) a 96-well elution plate for processed extracts; (4) a solvent reservoir and centrifuge balance for load equilibration; (5) an Acroprep™ Advance 30K Omega filtration plate positioned for integration with the PPU V4; (6) a Biomek i7 servo shuttle system for plate positioning; and (7) a moving tray enabling automated labware transfer to the PPU V4 module (Figure 8).

### 4.3. Preparation of Stock Solutions, Calibrators, and QC Samples

Primary stock solutions of CBD and 7-hydroxy-CBD were individually prepared in methanol at a concentration of 1 mg/mL. Working standard solutions were obtained by serial dilution of the corresponding stock solutions in methanol to yield final concentrations of 10, 20, 50, 100, 250, 500, and 1000 ng/mL for CBD, and 5, 10, 25, 50, 125, 250, and 500 ng/mL for 7-hydroxy-CBD. QC solutions were prepared independently from separate stock solutions to ensure validation integrity. Target concentrations for CBD were 35 ng/mL (low QC), 350 ng/mL (medium QC), and 750 ng/mL (high QC), while corresponding concentrations for 7-hydroxy-CBD were 17.5 ng/mL, 175 ng/mL, and 375 ng/mL, respectively. CBD-*d*_3_ was employed as the IS. The IS stock solution (100 μg/mL) was prepared in methanol, and a working solution was diluted to a final concentration of 170 ng/mL in methanol. All stock and working solutions were stored at −20 °C.

### 4.4. Manual Sample Preparation

The manual sample preparation procedure was conducted in accordance with the method previously described and validated by Franco et al. (2022) [36] and is briefly outlined herein for reference. Calibration standards and QC samples were prepared in human serum by mixing 75 μL of blank matrix with 75 μL of IS (CBD-*d*_3_, 170 ng/mL in methanol), 75 μL of a working solution containing CBD and 7-hydroxy-CBD, and 150 μL of methanol. For patient serum samples, the preparation involved the addition of 75 μL of serum, 75 μL of IS (CBD-*d*_3_, 170 ng/mL in methanol), and 225 μL of methanol. All samples were vortexed and centrifuged at 17,000× *g* for 10 min at 4 °C. An aliquot of 20 μL of the resulting supernatant was injected into the LC-MS/MS system for quantitative analysis.

### 4.5. Automated Sample Preparation

The calibrators and QC samples in human serum were prepared by mixing 75 μL of serum matrix with 75 μL of CBD and 7-hydroxy-CBD standard solution, 75 μL of IS (CBD-*d*_3_, 170 ng/mL in methanol), and 150 μL of methanol. Patient serum samples were processed by combining 75 μL of serum, 75 μL of IS (CBD-*d*_3_, 170 ng/mL in methanol), and 225 μL of methanol. All sample preparations were performed in a 96-well plate format. Following centrifugation with the integrated microplate centrifuge, sample purification was conducted using the automated PPU V4 system, which is incorporated within the Biomek i7 Hybrid Workstation. After centrifugation, the supernatant was transferred to Acroprep™ Advance 30K Omega filtration plate. Sample purification was facilitated by the PPU V4 system, operating with the following parameters: filter pressure set at 1500 mbar, clamp pressure at 3000 mbar, and a processing time of 10 min. The collection plate was subsequently inserted directly into the LC-MS/MS autosampler, where 20 μL of the sample was injected for analysis. To ensure compatibility with the Biomek i7 Hybrid Workstation and to achieve sufficient supernatant volume for downstream processing, the volumes of biological matrix, calibrators, IS, and extraction solvent were increased compared with the previously published manual protocol [36].

### 4.6. Clinical Samples and Comparison Between Manual and Automated Sample Preparation

Participants were recruited from the Child Neurology and Psychiatry Unit of the IRCCS Mondino Foundation (Pavia, Italy). This study protocol was approved by the local Ethics Committee (reference No.: 2023-3.11-112). Serum samples were obtained from six subjects clinically diagnosed with Lennox–Gastaut syndrome and treated with CBD (Table 6). Sampling was performed at steady-state conditions, 16 h after the last administered dose of CBD. The collected samples were immediately processed and stored at −20 °C until LC-MS/MS analysis. Statistical comparisons between manual and automated sample preparation methods were conducted using Passing–Bablok regression and Lin’s concordance correlation coefficient to assess systematic and proportional biases as well as overall agreement. LoA was calculated as the mean difference ± 1.96 times the SD of the differences, with the corresponding 95% CIs considered the predefined threshold for acceptable variability. The 95% CIs were computed for both the intercept and slope of the regression line. Furthermore, method agreement was evaluated using Bland–Altman analysis, which involved plotting the differences between paired measurements against their mean values. All analyses were performed using R version 4.2.3 (R Foundation for Statistical Computing, Vienna, Austria) within RStudio interface version 2024.4.2.764 (Posit team, Posit Software, PBC, Boston, MA, USA).

**Table 6:** Demographic and clinical characteristics of the six subjects with Lennox–Gastaut syndrome included in the analysis.

Patient Number	Age	Sex	Weigh, kg	Dose of CBD (mg/kg/day)	Concomitant ASMs
1	11	M	40	5	VGB, PB, CLB
2	19	M	46	8.7	VPA, CLB
3	19	F	47	10.6	CLB, RFN, LTG, PB
4	8	F	18	11.1	CLB, VPA, ETS
5	5	F	20	17.9	VPA, TPM, CLB
6	17	M	45	8.9	VPA

*Abbreviations: ASMs, antiseizure medications; CLB, clobazam; ETS, ethosuximide; F, female; LTG, lamotrigine; M, male; PB, phenobarbital; RFN, rufinamide; TPM, topiramate; VGB, vigabatrin; VPA, valproate.*

### 4.7. Online SPE

In the protocol described by Franco et al. (2022) [36], online SPE was implemented via a perfusion column (POROS R1, 2.1 × 30 mm i.d., 20 μm) to enhance sample cleanliness prior to LC–MS/MS analysis. In the present study, this column was used exclusively for serum samples processed through the manual sample preparation workflow. In contrast, the automated method integrated a high-efficiency sample cleanup step consisting of a 96-well Acroprep™ Advance 30K Omega filtration plate in combination with a PPU V4. This filtration-based purification approach proved sufficient to achieve the required analytical clarity, thereby eliminating the need for online SPE. The matrix removal process was highly efficient and reproducible, allowing for direct injection into the LC–MS/MS system without compromising analytical sensitivity or selectivity.

### 4.8. Method Validation

The LC–MS/MS analytical method employed in this study was previously developed and fully validated in compliance with EMA guidelines [36,37]. For the current automated sample preparation protocol, validation was conducted to assess performance characteristics relevant to the new pre-analytical workflow. Precision and accuracy were evaluated at four concentration levels: the LOQ, low QC, medium QC, and high QC. Intraday precision and accuracy were assessed using five replicates per concentration. Interday precision and accuracy were evaluated using ten replicates for the LOQ and twenty replicates for the QC samples. Precision was assessed using the coefficient of variation (CV%), with predefined acceptance criteria of ≤15% for QC samples and ≤20% for LOQ samples. Accuracy was determined by comparing the mean measured concentrations of QC and LOQ samples to their respective nominal values. Extraction recovery was calculated by comparing the peak areas of five replicates of LOQ and QC samples to those of non-extracted standard solutions prepared at equivalent concentrations of CBD and 7-hydroxy-CBD. The matrix effect was investigated using samples obtained from six distinct individuals. For each matrix, precision and accuracy were assessed by analyzing three replicates of both low- and high-concentration QC samples. This evaluation ensured that the automated system maintained analytical integrity and reproducibility throughout the sample preparation process.

## Data Availability

The raw data supporting the conclusion of this article can be found in Zenodo (10.5281/zenodo.15590614).

## Abbreviations

ASM	Antiseizure medications
CI	Confidence interval
CBD	Cannabidiol
CLB	Clobazam
CV	Coefficient of variation
EMA	European Medicines Agency
ETS	Ethosuximide
F	Female
HPLC	High-Performance Liquid Chromatography
IS	Internal standard
LC-MS/MS	Liquid chromatography coupled with tandem mass spectrometry
LLE	Liquid–liquid extraction
LoA	Limit of agreement
LOQ	Limit of quantification
LTG	Lamotrigine
M	Male
PB	Phenobarbital
PP	Protein precipitation
QC	Quality control
RFN	Rufinamide
SD	Standard deviation
SPE	Solid-phase extraction
TDM	Therapeutic drug monitoring
TPM	Topiramate
VGB	Vigabatrin
VPA	Valproate
